# Comparison of Cryptococcal Antigenemia between Antiretroviral Naïve and Antiretroviral Experienced HIV Positive Patients at Two Hospitals in Ethiopia

**DOI:** 10.1371/journal.pone.0075585

**Published:** 2013-10-04

**Authors:** Tafese Beyene, Yimtubezinash Woldeamanuel, Daniel Asrat, Gonfa Ayana, David R. Boulware

**Affiliations:** 1 Department of Biomedical Sciences; Asella School of Health Sciences, Adama Science and Technology University, Asella, Ethiopia; 2 Department of Microbiology, Immunology and Parasitology; College of Health Sciences, Addis Ababa University, Addis Ababa, Ethiopia; 3 Regional Laboratories Capacity Building Directorate, Ethiopian Health and Nutrition Research Institution (EHNRI), Addis Ababa, Ethiopia; 4 Division of Infectious Diseases and International Medicine, Department of Medicine, University of Minnesota, Minneapolis, Minnesota, United States of America; University of Malaya, Malaysia

## Abstract

**Background:**

Cryptococcal meningitis is a major cause of HIV/AIDS-related deaths in Africa. Cryptococcosis is a neglected killer. However, meningitis can be prevented by early cryptococcal antigen (CrAg) screening and preemptive antifungal treatment during a prolonged period of detectable, subclinical infection. We determined the prevalence of cryptococcal antigenemia in comparison to CD4 count and clinical symptoms.

**Methods:**

We surveyed 254 consenting HIV-infected participants to obtain demographic information and clinical history. Serum CrAg was measured by latex agglutination at two sites in the Oromia region of Ethiopia among all persons receiving a CD4 count.

**Results:**

Of the 254 participants, 127(50.0%) were ART-naïve, 121(47.6%) were ART-experienced, and 6(2.4%) were ART-defaulters. The prevalence of cryptococcal antigenemia was 10.2% overall being 14.2% among ART-naive, 4.1% among ART-experienced, and 50% (3/6) among ART-defaulters, irrespective of CD4 count. Cryptococcal antigenemia was more frequently detected from ART-naïve patients (p = 0.012) and ART-defaulters (p = 0.001) compared with ART-experienced. Serum CrAg positivity was 20.9% in persons with CD4≤150 cells/µL, 12.2% in 151–200 cells/µL, 5.8% among 201–350 CD4/µL, and none above 350 cells/µL. Potential meningitis symptoms were common in the outpatient cohort irrespective of CrAg-status, with only fever and altered mental status statistically more common in CrAg-positive compared to CrAg-negative persons (P<0.05), yet no symptom had a positive predictive value >33%.

**Conclusion:**

We report a 20.9% cryptococcal antigenemia prevalence among those with CD4+ T cells count ≤150 cells/µL, irrespective of ART status, with even higher CrAg prevalence in ART-naïves and ART-defaulters. These groups are target populations for CrAg screening at entry into HIV care.

## Introduction

Cryptococcal meningitis is a major cause of death among HIV/AIDS patients accounting for 20–25% of AIDS-related mortality in Africa [1]. Where effective antiretroviral therapy (ART) is universally available, the incidence of cryptococcosis, along with other opportunistic infections (OIs), has decreased [2]. Yet, cryptococcosis is the most common cause of adult meningitis in Central, Eastern, and Southern Africa [3]–[6]. Cryptococcus is a neglected killer [7].

High proportions of cryptococcal deaths are potentially preventable by screening for cryptococcal antigen (CrAg) at entry into HIV programs and providing pre-emptive antifungal treatment before symptoms of meningitis develop [8]–[10]. CrAg is detectable in blood weeks to months prior to the development of overt clinical symptoms [11]. This prolonged subclinical period of asymptomatic infection presents an opportunity to identify persons with asymptomatic or early disease.

ART alone is insufficient treatment for CrAg-positive HIV-infected persons [10], [12], [13]. Among those with asymptomatic cryptococcal antigenemia, these persons are at high risk of developing symptomatic CM and/or death despite receiving ART [10], [12]. Proactive management is needed for HIV-infected patients with CD4+ T-cell count ≤100 cells/µL [10], and antifungal treatment is recommended for all patients with cryptococcal antigenemia before ART initiation [14], [15]. Primary fluconazole prophylaxis is another prevention strategy [16]; however CrAg screening and preemptive treatment is recommended as a more cost-effective strategy [14], [17].

The present study sought to determine the prevalence of cryptococcal antigenemia among HIV-infected individuals in Ethiopia to determine if pre-ART CrAg screening should be implemented as routine policy. We compared the prevalence of cryptococcal antigenemia by CD4 count and between ART-experienced and ART-naive patients in two hospitals in Ethiopia.

## Methods

This research was ethically approved by the Department Research and Ethical Review Committee (DREC) of Department of Microbiology, Immunology and Parasitology, College of Health Science, Addis Ababa University and Oromia Health Bureau. Adult participants were informed about the study and their written consent taken, while for children their parents or guardians were informed about the study and their written consent taken. All laboratory results were communicated to the ordering physicians to enable clinical management.

A prospective cross-sectional study was conducted from December, 2011 to October, 2012 at Asella Teaching Hospital of Adama Science and Technology University and Adama Hospital. Both referral hospitals are in the Oromia region of southeastern Ethiopia approximately 175 and 100 kilometers from Addis Ababa, respectively, serving a catchment population of 4 million and 3.5 million people, respectively. Both hospitals provide general outpatient and inpatient services.

The study participants were all HIV-infected individuals who enrolled in the ART clinics, provided informed consent, and for whom CD4+ T-cells were tested during the study period from both outpatients (n = 220) and hospitalized patients undergoing provider initiated HIV counseling and testing (n = 34). Exclusion criteria included those had received amphotericin or fluconazole treatment in the last six months.

Serum samples were tested with the Remel Cryptococcal Antigen Test Kit (Remel, London, UK), according to the manufacturer's instructions. The serum was treated with protease enzyme, heating at 100°C for five minutes, cooling to room temperature, and then mixing with latex reagent. Any clumping or clearing of the test latex immediately after the 5 minute rotation indicated the specimen contained cryptococcal antigen. Positive and negative controls were run with each sample. Positive specimens had titers performed by two-fold serial dilutions. Cultures were performed on the serum specimens with the sera of the CrAg positive samples being re-centrifuged and the sediment directly inoculated on Sabouraud dextrose agar slants tubes with gentamicin (40 mg/mL) and incubated at 37°C for 2 weeks. CD4^+^ T-cells were measured using FACSCalibur Flow Cytometry (BD, San Jose, USA).

### Statistical Analysis

Analysis was primarily descriptive with characteristics summarized by mean, median (range), and frequencies (%) as relevant. Fisher exact Chi-square test compared categorical variables for statistical significance. To assess for risks of cryptococcal antigenemia, logistic regression compared demographics, HIV parameters, and clinical symptoms by univariate and then multivariable analysis using logistic regression. Epi-info 3.5.3 was used for data entry, and SPSS 19.0.1 (IBM) was used for statistical analysis. P-values <0.05 were considered statistically significant.

## Results

Overall, 254 HIV-infected persons from Asella teaching hospital (n = 102) and from Adama hospital (n = 152) were enrolled. Of the 254 participants, 127(50.0%) were ART-naïve, 121(47.6%) were ART-experienced, and 6(2.4%) had defaulted from ART (“ART-defaulters”). The median age was 33 years, with 79% (202/254) between 16–45 years old. Demographics are presented in **Table 1**.

**Table 1:** Demographics of Cohort.

Variables	Frequencies	Percent (%)
**Sex**		
Male	115	45.3
Female	139	54.7
**Age in years**		
<16	15	5.9
16–30	92	36.2
31–45	110	43.3
>45	37	14.6
**Residence**		
Urban	160	63.0
Rural	94	37.0
**ART status**		
ART-Experienced	121	47.6
ART-Naive	127	50.0
ART-defaulted	6	2.4
**WHO Clinical Stages**		
Stage I or II	70	27.6
Stage III	92	36.2
Stage Iv	92	36.2
**Patients**		
Outpatients	220	86.6
Inpatients	34	13.4
**Total**	**254**	**100%**

The overall prevalence of cryptococcal antigenemia was 10.2% (26/254) with 14.2% (18/127) among ART-naive, with 4.1% (5/121) among ART-experienced and with 50% (3/6) among ART-defaulters. Cryptococcal antigenemia was more frequently detected from ART-naïve patients (p = 0.012) and ART-defaulters (p = 0.001) than in persons receiving ART (**Table 2**). As expected, CrAg positivity was associated with greater immunosuppression; however, unexpectedly a significant number of persons with CD4>100 cells/µL were positive for serum CrAg. Among persons with CD4≤150 cells/µL, CrAg positivity was 20.9% (18/86). Serum CrAg was positive in 12.2% among CD4 151–200 cells/µL, 5.8% among 201–350 cells/µL, and none above 350 cells/µL (**Table 3**). Among outpatients only, the CrAg prevalence was 13% (13/100) in ART-naïve persons, 4.3% (5/117) in ART-experienced, and 3 of 3 in ART-defaulters among all participants irrespective of CD4 count. In outpatient ART-naïve person with CD4<200 cells/µL, the CrAg prevalence was 29% (12/41; 95% CI (16%–45%). Out of 26 CRAG positive persons, and 11(42%) had CrAg titers between 1∶4 and 1∶128, 9 (35%) had CrAg titers between 1∶256 and 1∶1024, and 6 (23%) had CrAg titers >1∶1024. Four persons had blood cultures which grew *Cryptococcus neoformans.*

**Table 2:** Cryptococcal antigenemia and Antiretroviral Therapy (ART) status.

ART status	CrAg Test		Total (%)	P value
	Positive (%)	Negative (%)		
On ART	5 (4.1%)	116 (95.9%)	121 (47.6%)	Reference
ART-naïve	18 (14.2%)	109 (85.8)	127 (50.0%)	0.012
ART-defaulter	3 (50%)	3 (50%)	6 (2.4%)	0.001
**Total**	**26 (10.2%)**	**228 (89.8%)**	**254 (100%)**	

P-value determined by Fisher’s exact test using those on ART as the reference group.

**Table 3:** Association of Serum CrAg Positivity with Degree of Immunosuppression.

Variables	N	CrAg+N(%)	Odds Ratio(95% CI)
**CD4+T-cells/µL**			
≤150	86	18 (20.9%)	4.0 (1.1–19.6)
≤50	32	8 (25.0%)	5.4 (1.2–28.8)
51–100	27	5 (18.5%)	
101–150	27	5 (18.5%)	
151–200	41	5 (12.2%)	
201–350	52	3 (5.8%)	
>350	75	0 (0%)	
**Total Lymphocyte Count**			
≤1000/µL	53 (20.9)	12 (22.6%)	3.9 (1.7–9.1)
>1000/µL	201 (79.1)	14 (7.0%)	
**WHO Clinical Staging**			
Stage I or II	70 (27.6)	0 (0.0%)	
Stage III	92 (36.2)	7 (7.65)	5.7 (0.7–125.9)
Stage IV	92 (36.2)	19 (20.7%)	18.0 (2.4–369.6)
**Total**	**254 (100)**	**26 (10.2%)**	

In assessing other risk factors for CrAg positivity beyond CD4 and ART status, gender, marital status, and residence locale (urban vs. rural) were unrelated. Of those 26 who were CrAg+, many had symptoms. Common symptoms included headache (73%, n = 19), fever (62%, n = 16), cough (54%, n = 14), skin lesions (38.5%, n = 10), seizures (11.5%, n = 5), and vomiting (11.5%, n = 3) at data collection time (Table 4). In restricting to only outpatients, symptomatic complaints were common irrespective of CrAg status. For example, 72% of CrAg+ had headache, but 41% of CrAg-negative outpatients also had headaches, equating to a positive predictive value of 16%. Among the ART-naive outpatient population, headache, stiff neck, cough, seizure, and vomiting were not statistically increased in CrAg+ compared to CrAg-negative. Fever and altered mental status were statistically more common in CrAg+ compared to CrAg-negative. However, the positive predictive value for any symptom was poor (<33%), thus CrAg screening based on symptoms would not increase the efficacy and would be less efficient than reflex screening based on CD4 count.

**Table 4:** Clinical manifestation associated with cryptococcal antigenemia.

Clinicalmanifestations	CrAg positiveN = 26	CrAg negativen = 228	Crude Odds Ratio(95%CI)	Multivariate AdjustedOR (95%CI)	PositivePredictive Value
**Headache**	19(73%)	97 (43%)	3.6(1.4–8.8)	1.8(0.7–5.2)	16%
**Fever**	16(62%)	56 (25%)	4.9(2.1–11.4)	**3.1(1.2–8.1)**	22%
**Cough**	14(54%)	77 (34%)	2.3 (1.0–5.2)	1.4(0.6–3.7)	15%
**Skin lesion**	10(38%)	71 (31%)	1.4(0.6–3.2)		12%
**Altered mental Status**	8(31%)	18 (7.9%)	5.2 (2.0–13.6)	**3.1(1.1–9.4)**	31%
**Seizure**	5(19%)	13 (5.7%)	3.9 (1.3–12.1)	2.1(0.5–9.6)	28%
**Stiff neck**	4(15%)	8 (3.5%)	5.0(1.4–17.9)	1.4(0.2–8.4)	33%
**Vomiting**	3(12%)	14 (6.1%)	2.0 (0.5–7.5)		18%

## Discussion

**Figure 1:**
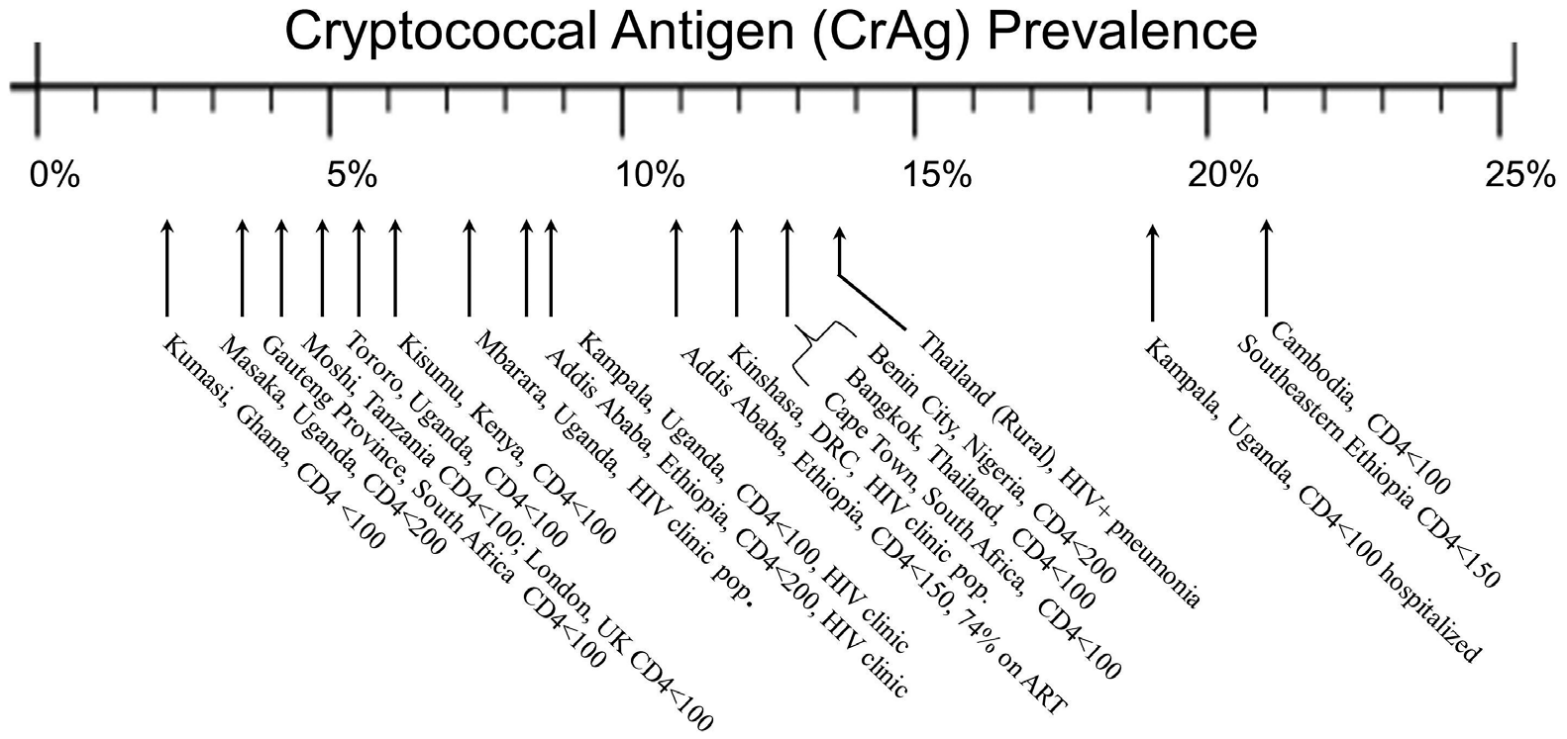
Cryptococcal Antigen Prevalence in Reported Cohorts. The prevalence of cryptococcal antigenemia was 21% among those with CD4 count <150 cells/µL, irrespective of ART status, in this two-site study in southeastern Ethiopia. This prevalence is similar as in Cambodia 21.0% [18] but higher than reported from Addis Ababa, Ethiopia [26], Uganda [10], 13,19,20, Thailand [9], Ghana [21], Tanzania [22], Democratic Republic of the Congo (DRC) [23], Cape Town, South Africa [12], Nigeria [24], Vietnam [27], and in a pneumonia cohort from Thailand [25].

Our findings indicate that the overall prevalence of cryptococcal antigenemia was 10.2% in Ethiopia among HIV-infected persons receiving a CD4 count measurement. Among outpatient ART-naïve persons, the CrAg prevalence was 13% irrespective of CD4 count, and among outpatient ART-naïve with CD4<200 cells/µL, the CrAg prevalence was 29% (95% CI: 16%–45%). Among all persons with a CD4 count <150 cells/µL, irrespective of ART status, the serum CrAg prevalence was 21% (95% CI: 12.9–31%). This CrAg prevalence is similar as reported in Cambodia 21% [18] but higher than reported elsewhere [9], [10], [12], [13], [19]–[27] (**Figure 1**).

The presence of CrAg in peripheral blood presents an important opportunity to identify persons several weeks prior to the development of obvious symptoms of meningitis with early or asymptomatic disease [8], [28]. Cryptococcal-related deaths can be prevented by screening for CrAg at entry into HIV programmes and providing pre-emptive anti-fungal treatment before symptoms of meningitis develop. Treatment of antigenemia is a fraction (<10%) of the cost of treating cryptococcal meningitis [8], [27], [29], [30], and long term outcomes are better [31]. However, in Ethiopia, to our knowledge, no HIV clinic has implemented pre-ART CrAg screening as routine policy. Therefore we conducted the present study to identify the target populations and to advocate the use of the early screening CrAg in HIV clinics in the country. Ethiopian cryptococcal data are limited. The prevalence of cryptococcal meningitis accounted for 8.2% of admisisons of HIV-infected persons to Gonder hospital [32] and CSF India ink was positive meningitis account for 7% of meningitis cases at Tikur Anbessa Hospital in 2001, long before the peak of the HIV epidemic [33]. One recent 2013 cross-sectional study conducted in Addis Ababa, reported an 10% CRAG prevalence among Ethiopians with a CD4<150 cells/µL; however, 74% of the cohort was receiving ART for an average of 3-years [26]. This lower prevalence than in our study may reflect a survival bias as the unmasking of cryptococcosis and death typically occurs within the first 4 months of ART for those pre-ART CrAg-positive with titers >1∶8 [10], [12]. As the 6-month survival after developing cryptococcal meningitis is typically <50% in Africa, having received 3-years of ART likely does incorporate a survival bias, which may be an important reason why the reported prevalence rates in two regions of Ethiopia appear so different. Nevertheless, whether the “true” prevalence is 10% or 20% or somewhere in between, this markedly high CrAg prevalence calls for immediate implementation of pre-ART CrAg screening in Ethiopia HIV programmes, in line with 2013 PEPFAR technical guidance [34].

Our observation was that 23% of cryptococcal antigenemia positive patients (5 among ART-naïve and 1 among ART-defaulters) had high antigen titers (>1∶1024). However, all the persons currently receiving ART had lower CrAg+ titers <1∶512. Performing a CrAg titer for positive results is important to stratify risk [35], [36], yet future studies are needed to better define risk strata. In symptomatic patients or when there is a high CrAg titer, we would recommend that LP be performed to exclude CNS involvement. In low CrAg antigen titers (<1∶256), fluconazole treatment alone *may* be adequate in asymptomatic patients without a LP [8], [12], [35]. Further research is necessary to determine when lumbar punctures must be performed in order to guide clinical care. Lack of the ability to perform a lumbar puncture in a rural health care center should not be a reason to not implement pre-ART CrAg screening; however, triage of CrAg-positive may then be necessary.

The majority of CrAg-positive patients had some symptoms, and few were completely asymptomatic. Headache was the most common symptom, being present in 72% of CrAg-positive outpatients. Yet, headache was overall a very common symptom with 48% of the entire HIV clinic population reporting symptoms of headache. It would be difficult to perform lumbar punctures on every person with a headache, [37] thus CrAg titer may be one method to stratify risk; however, such a strategy requires further assessment and validation.

Our data show that most of the patients (81%) with cryptococcal antigenemia were among those: 1) newly diagnosed with HIV infection in outpatient setting, 2) diagnosed via provider initiated HIV counseling and testing as inpatient, or 3) diagnosed among persons who had defaulted from ART and were re-entering HIV care. Among the inpatients, most patients were unaware of their HIV-serostatus and came to the hospitals when they began to have symptomatic illness. Among the 26 cryptococcal antigenemia positive patients, 69% were ART-naïve. This is similar to Cape Town where 54% patients presented with incident cryptococcal antigenemia prior to ART initiation [12].

Among those CrAg-positive, 88.5% had CD4+ T-cells count ≤200 cells/µL. These results are in agreement with the findings from Uganda, Tanzania, and South Africa where >90% had CD4+ T cells counts ≤200 cells/µL [11], [38], [39]. Another finding of the present study is among those with CD4+ T cells count between 201–350 cells/µL, 5.8% were CrAg+. In contrast, in Thailand and in Tanzania, no persons were CrAg+ and with a CD4 count >200 cells/µL [9], [40]. However, antigen positivity above 200 CD4 cells/µL is in line with the report of 2% in Uganda [41]. The clinical relevance of CrAg positivity among persons with CD4 counts >100 cells/µL is currently doubtful [10].

The majority of deaths that occur in persons living with AIDS in Ethiopia are in the first four months of ART [42]. Since the prevalence of cryptococcal antigenemia is high among ART-naïve persons with low CD4+ T-cell counts, unmasking of cryptococcosis is likely a significant contributor to early mortality. Among untreated CrAg positives, 5–7 fold higher mortality occurs in those not receiving preemptive antifungal treatment [12]. Unmasking cryptococcomas are ring-enhancing on CT scanning, and thus can be commonly mistaken for toxoplasmosis and/or CNS-lymphoma. These diagnoses should have cryptococcosis excluded, as cryptococcomas are treatable.

We found the numbers of ART-naive persons not yet accessing HIV care until they presented with low CD4+ T-cells counts remained high, and cryptococcal antigenemia was predominantly diagnosed among this group. With this late presentation to HIV care, these persons are at high risk of OIs, and the *Cryptococcus* burden is likely to continue undiminished in many areas [43], [44].

Cryptococcal management is urgently needed by facilitating earlier diagnosis and treatment. A prospective study conducted in Uganda indicated CrAg screening is both cost-effective and affordable in resource-limited settings [10], with similar findings from Vietnam [27]. This is possible at the same time when CD4+ T-cells count is ordered by screening the remaining plasma for CrAg in low CD+ T-cells count (≤100 or <150 cells/µL). First of all, CrAg testing t assists to identify asymptomatic cryptococcal antigenemia and preemptive treatment reduces the chance of developing meningitis [10], [45]. Secondly, treatment of antigenemia is less expensive, less toxic than amphotericin B, and results in better 5-year survival than treating symptomatic cryptococcal meningitis [8], [29], [31]. Generally, early management of cryptococcal infection and access to ART offers the possibility of a good long term prognosis and improves patients’ survival [31], [46].

Limitations of the current cross-sectional study include the lack of specific outcome data from this multisite study; however, outcomes would be expected to be similar as previously reported in Uganda and South Africa [10], [12], [13]. Second, these prevalence data reflect the prevalence of CrAg among those having a CD4 test ordered. This may be different than the prevalence among all clinic attendees; however, this prevalence is informative from a laboratory perspective, in considering reflex lab-based testing, which we would recommend.

In the present study, we found an overall prevalence of 10.2% cryptococcal antigenemia in HIV-infected patients with higher prevalence in ART-naïve (14%), ART-defaulters (3 of 6), and among all with CD4+ T-cells count ≤150 cells/µL (21%). The standard of care should be altered such that whenever a CD4 count is <150 cells/µL that the laboratory reflexive also tests CrAg. Such lab “reflex CrAg testing” has been advocated for ART naïve populations [8], [35]. This study demonstrates that among persons with low CD4 counts, ART-status becomes less important. As laboratory staff often may not be aware of ART status, this detail may be unnecessary for implementing CrAg screening. A new CrAg lateral flow immunochromatographic assay (LFA) (Immy, Inc., Norman, Oklahoma, U.S.) is now available which has excellent performance on plasma [47], [48], thus reflex testing is possible on left over plasma remaining from CD4 testing. The CrAg LFA is a point-of-care test requiring no lab infrastructure, room temperature storage, and cost is $2/assay for resource-limited countries ($5 for high-income countries). Once early HIV testing, linkage-to-care, retention-in-care, and access to ART are universally available, CrAg screening will be unnecessary. Yet in order to prevent unnecessary deaths now, CrAg screening should be immediately implemented in persons presenting with low CD4 counts, as recommended by WHO guidelines and PEPFAR technical guidance [14], [34]. In Sub-Saharan Africa, reflexive CrAg testing after CD4 counts should start immediately.
